# Injury epidemiology at the 2022 and 2023 senior wrestling world championships

**DOI:** 10.1186/s40621-026-00697-y

**Published:** 2026-07-31

**Authors:** Marton Jasz, Babak Shadgan, Karsa Molnar, Peter Rein, Radivoj Filipov, Szabolcs Molnar

**Affiliations:** 1 Budapesti Dr Manninger Jeno Baleseti Kozpont, Budapest, Hungary; 2 Hungarian Wrestling Federation Medical, Prevention & Anti-Doping Commission, Budapest, Hungary; 3 Department of Orthopaedics, University of British Columbia, Vancouver, Canada; 4 Medical, Prevention and Anti-Doping Commission, United World Wrestling, Corsier-sur-Vevey, Switzerland; 5 Department of Orthopaedics, Central Hospital of Northern Pest - Military Hospital, Budapest, Hungary; 6 Department of Orthopedics, Semmelweis University, Budapest, Hungary; 7 Disease Control and Prevention, Institute of Public Health of Zrenjanin, Zrenjanin, Serbia; 8 Department of Orthopedics and Trauma Surgery, University of Debrecen, Debrecen, Hungary

**Keywords:** Wrestling, Sports injuries, Injury epidemiology, Injury surveillance, Incidence, Risk factors, Athlete safety, Combat sports

## Abstract

**Background:**

Wrestling World Championships represent the highest level of competition in the sport, featuring hundreds of elite athletes across three styles and ten weight classes. This study aims to analyze injury patterns and risk factors in senior-level wrestling.

**Methods:**

Injury data were recorded during the 2022 and 2023 Senior Wrestling World Championships. Athlete gender, wrestling style, injury severity, type, and location were documented.

**Results:**

Among 1,686 competitors and 3,882 athletic exposures (AEs), 107 injuries were recorded, yielding an overall injury incidence rate of 27.56 per 1,000 AEs. Injury incidence rates per style were 25.55/1,000 AEs in Greco-Roman, 34.31/1,000 AEs in Freestyle, and 21.22/1,000 AEs in Women’s Wrestling. The most common injuries were strains (33.6%, 9.27/1,000 AEs) and superficial skin injuries (24.3%, 6.70/1,000 AEs). Injuries predominantly affected the limbs (45,79%) and head-neck region (45,79%). Injuries in the face-neck region were mostly minor, whereas limb injuries had a higher proportion of more severe cases.

**Conclusions:**

The injury patterns observed are consistent with prior data from high-level wrestling competitions. The findings of our study are consistent with previously reported values regarding the incidence and severity of injuries. Notably, limb injuries were associated with greater severity.

## Background

Wrestling is among the oldest combat sports, with origins tracing back to ancient civilizations. It has been a core Olympic discipline since the inception of both the Greek and modern Olympic Games. Currently, Olympic wrestling comprises three styles: Greco-Roman (GR), in which attacks below the waist are prohibited; Freestyle (FS), where leg attacks are permitted; and Women’s Wrestling (WW), which follows FS rules. Senior matches last 2 × 3 min with a 30-second break between rounds [1]. 

The World Championships (WCh) represent the highest level of competition outside the Olympic Games. Each nation can enter one athlete per style and weight category, competing across ten weight classes per style. Despite wrestling’s intense physicality, its injury rate is comparable to that of other Olympic sports [2–4]. The Medical Commission (MC) of United World Wrestling (UWW) continuously monitors injury trends to develop preventative measures. Various papers claim that previous rule changes, including same-day weigh-ins, protocols for strangulation injuries, guidelines for bleeding time, and medico-referee sign language, have successfully reduced the incidence of injuries [5, 6]. 

A standardized injury registration protocol is applied at every UWW competition. UWW medical professionals document injuries on paper and later input them into an online surveillance system [7, 8]. The current data collection framework aligns with the 2020 International Olympic Committee (IOC) recommendations, capturing variables such as gender, wrestling style, severity, injury type, anatomical location and athletic exposures (AEs) [9]. One AEs equals one athlete’s participation in one match/duel/event, etc. In wrestling one match therefore equals 2 AEs.

This study aims to create a descriptive epidemiological summary of two consecutive World Championships for future reference.

## Methods

Study participants included elite wrestlers who competed in the 2022 and 2023 Senior Wrestling World Championships in Belgrade. The unit of analysis was the athlete-appearance, defined as a single athlete participating in a single championship year. Athletes who competed in both years were non-identifiable and contributed separate athlete appearances for each competition year.

Any injury event requiring intervention by the on-site medical team was classified as such and subsequently documented as described earlier, following the standardized UWW-MC protocol. Recorded variables included gender, nationality, wrestling style, weight category, injury date, severity, mechanism, type, and location. Severity classification is based on the level of medical intervention required during the competition, as after the competition, follow-up was not carried out, the level of the on-site medical care can be cleared easily. Severity was classified into 3 + 1 categories. Mild injuries were those that could be managed directly on the mat without necessitating further evaluation. Moderate injuries required additional examination or treatment at the competition venue but did not prevent the athlete from continuing participation. Severe injuries led to match termination or necessitated hospital transfer for specialized care. The most critical category, catastrophic injuries, encompassed life-threatening conditions that posed an immediate and serious risk to the athlete’s health. Based on our analysis, we excluded the severity category “catastrophic” due to the lack of such cases.

For statistical analysis, we used Fisher’s Exact test to compare injury incidence across genders, wrestling styles, qualification rounds, and final rounds, and to compare injury severity across styles and anatomical regions. For each test, we used the /1000-corrected AEs incidence. The Data collection and handling adhered to UWW regulations and GDPR guidelines.

## Results

A total of 1,686 athletes (2022: 745; 2023: 941) participated in 1,941 matches, totaling 3,882 AEs (GR: 1,370; FS: 1,428; WW: 1,084). There were 114 recorded injuries (GR: 37; FS: 53; WW: 24). Warm-up and training injuries accounted for 7 non-bout cases, resulting in 107 injuries during matches, yielding an overall incidence of 27.56 injuries per 1,000 AEs. Incidence rates and proportion of all injuries in per style were 25.55/1,000 AEs, 32.7% in GR, 34.31/1000 AEs, 45.8% in FS, and 21.22/1,000 AEs, 21.5% in WW (Table 1).

**Table 1:** The incidence of injuries at the 2022 and 2023 Senior Wrestling World Championships, categorized by severity and wrestling styles

	Mild	Moderate	Severe	All	Incidence (/1,000 AEs)
**GR**	23	4	8	35 *(32.71%)*	25.55
**FS**	31	13	5	49 *(45.79%)*	34.31
**WW**	8	12	3	23 *(21.5%)*	21.22
**All**	62	29	16	107 *(100%)*	27.56
**Incidence (/1**,**000AEs)**	15.97	7.47	4.12		

We compared injury data by gender and wrestling style. WW had 23 injuries in 1084 AEs, resulting in an incidence of 21.22/1000 AEs, while male wrestlers suffered 84 injuries in 2798 AEs, resulting in an incidence of 30.02/1000 AEs. Gender did not significantly impact injury incidence (OR 0.84, 95% CI 0.52–1.35, *p* = 0.562), and there was no significant difference between male styles (OR 1.37, 85% CI 0.88–2.14, *p* = 0.178). Final matches (semifinals, finals, and bronze medal bouts) had injuries more often (23 injuries in 600 AEs, 36.7/1,000 AEs) than qualification rounds (85 injuries in 3283 AEs, 25.9/1,000 AEs); however, the difference is not statistically significant (OR 1.43, 95% CI 0.89–2.31, *p* = 0.137). By severity, 57.95% of injuries were mild, 27.1% moderate, and 14.95% severe, with no catastrophic injuries recorded.

**Fig. 1:**
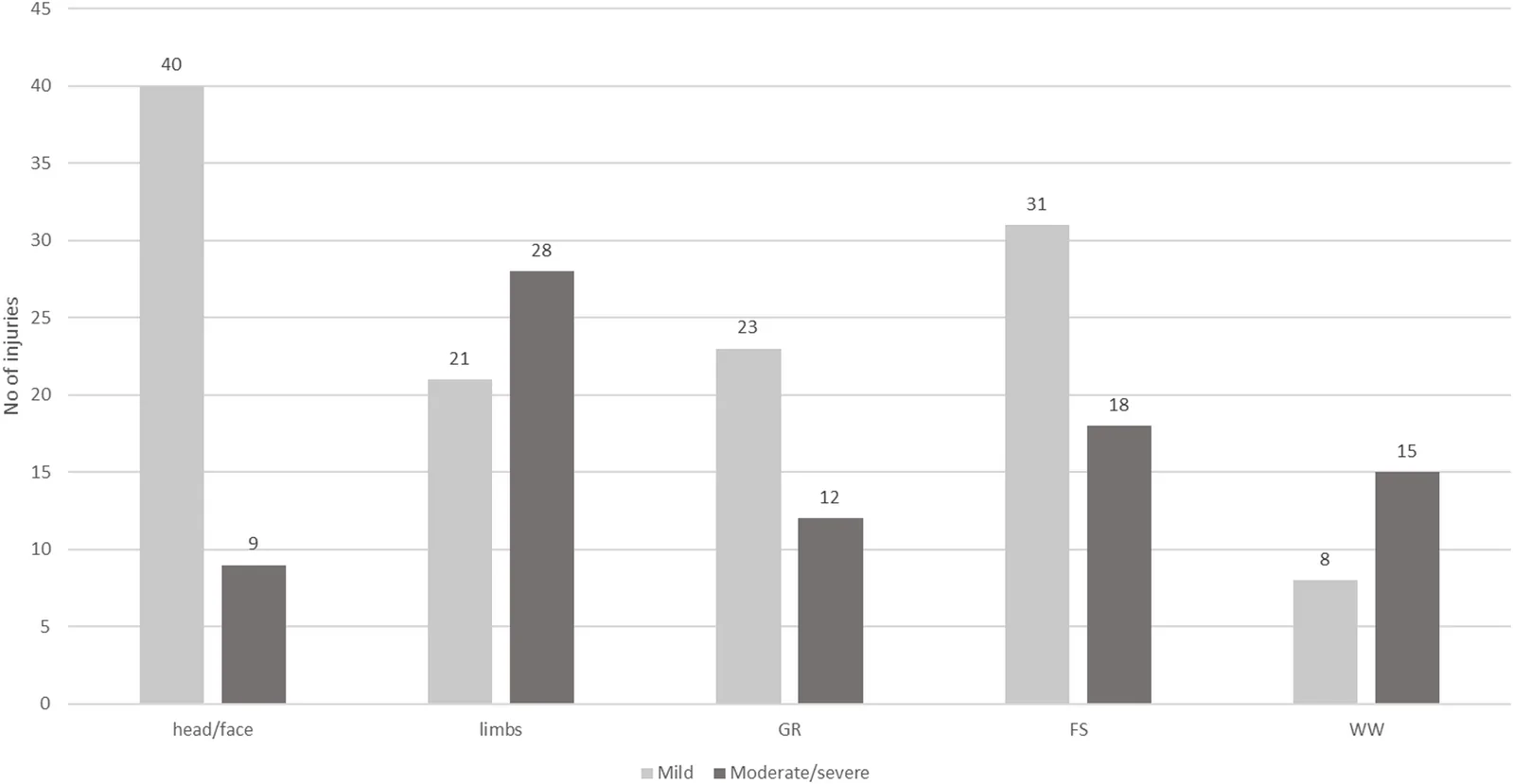
Number of mild and moderate/severe injuries in different anatomical regions and styles

The ratio of mild to moderate/severe injuries varied by gender and wrestling style. In GR, there were 23 mild injuries (16.96/1,000 AEs) and 12 moderate/severe injuries (8.76/1,000 AEs). In FS, 31 injuries were mild (21.7/1,000 AEs), and 18 were moderate/severe (12.61/1,000 AEs). Women’s Wrestling recorded 8 mild injuries (7.38/1,000 AEs) and 15 moderate/severe injuries (13.84/1,000 AEs). In general, there were 54 mild injuries (19.30/1,000 AEs) and 30 moderate to severe injuries (10.72/1,000 AEs) when considering both GR and FS (Diagram 1). Our analysis indicates that factors such as gender and injury location may affect the severity of injuries. Injuries in the facial region were more likely to be mild, whereas limb injuries were more often moderate/severe (OR 5.93, 95% CI 2.37–14.84 *p* < 0.001) (Fig. 1). Female athletes also exhibited a higher proportion of moderate/severe injuries than males (OR 3.38 95% CI 1.28–8.88 *p* = 0.016). Between the two male styles, GR and FS, neither was associated with an elevated risk for more severe injuries (OR1.11, 95% CI: 0.45–2.76, *p* = 1).

The most common injury type was ligament/joint strains (33.6%, 9.27/1,000 AEs), followed by superficial skin injuries (24,3%, 6.7/1,000 AEs), muscle injuries (9.35%, 2.58/1,000 AEs), bruises (8.41%, 2.32/1,000 AEs), bleeding (6.54%, 1.8/1,000 AEs), gum/lip injuries (6.54%, 1.8/1,000 AEs), and concussions/strangulation injuries (4.67%, 1.29/1,000 AEs). Strangulation injuries had an incidence of 0.8/1,000 AEs, all of which were of moderate severity. Joint dislocations occurred in 4 cases (1.03/1,000 AEs), bone injuries in 2 cases (0.52/1,000 AEs), and we registered an acute abdominal case, resulting in an intestinal perforation in 1 case (0.26/1,000 AEs) (Table 2).

**Table 2:** The number, percentage, and incidence of injuries categorized by injury type

Type of injury	Number	Percentage	Incidence (/1,000AEs)
Ligament strains	36	33.6%	9.27
Superficial skin injuries	26	24.3%	6.70
Muscle injuries	10	9.3%	2.58
Bruises	9	8.4%	2.32
Bleeding	7	6.5%	1.80
Gum or lip injuries	7	6.5%	1.80
Concussions, strangulation	5	4.7%	1.29
Joint dislocation	4	3.7%	1.03
Other bone injuries	2	1.9%	0.52
Other (faint due to exhaustion)	1	0.9%	0.26

Injury distribution by anatomical region revealed that most injuries affected the head and face (45.79%, 12.62/1,000 AEs), with 44 cases involving the face (11.33/1,000 AEs) and 5 affecting other parts of the head (1.29/1,000 AEs). Lower limb injuries accounted for 27.1% (7.47/1,000 AEs), while upper limb injuries comprised 18.69% (5.15/1,000 AEs) of all injuries. Trunk (spine and chest) injuries were recorded in 8 cases (7.48%, 2.06/1,000 AEs), and one abdominal injury was noted (0.9%, 0.26/1,000 AEs). The knee was the most affected joint (14.02%, 3.86/1,000 AEs), followed by the shoulder (11.21%, 3.09/1,000 AEs). Other commonly injured areas included the elbow and lower leg/ankle (each 7.48%, 2.06/1,000 AEs), hip/groin and thigh areas (each 2.8%, 0.77/1,000 AEs), cervical and lumbar spine (1.87% each, 0.52/1,000 AEs), and ribs (3.74%, 1.03/1,000 AEs) (Table 3).

**Table 3:** The number, percentage, and incidence of injuries categorized by injury region

Region	Number of injuries	Percentage of all	Incidence (/1,000AEs)
Head and face	49	45.8%	12.63
Face	44	41.1%	11.33
Head	5	4.7%	1.29
Lower limb	29	27.1%	7.47
Knee	15	14.%	3.86
Ankle, lower leg	8	7.5%	2.06
Hip, groin	3	2.8%	0.77
Thigh	3	2.8%	0.77
Upper limb	20	18.7%	5.15
Shoulder	12	11.2%	3.09
Elbow	8	7.5%	2.06
Trunk	8	7.5%	2.06
Cervical spine	2	1.9%	0.52
Lumbar spine	2	1.9%	0.52
Chest	4	3.7%	1.03
Abdomen	1	0.9%	0.26

## Discussion

This study provides a comprehensive analysis of injury incidence, severity, and distribution in elite-level wrestling, reinforcing prior findings while highlighting injury patterns among different wrestling styles. The injury incidence of 27.56 injuries per 1,000 athletic exposures (AEs) observed in the two wrestling World Championships aligns with prior studies, which report a range of 16.3 to 69.5 injuries per 1,000 AEs [10]. The injury rates differed by wrestling style, with distribution patterns similar to those reported by Shadgan et al. in the 2016 Rio Olympics: GR at 32.7% vs. 36.4%, FS at 45.8% vs. 40.9%, and WW at 21.5% vs. 22.7% [11]. Previous meta-analyses indicate GR and FS injury incidences of 26.3 and 35.8 injuries per 1,000 AEs, respectively, which corroborate our findings of 25.55 per 1,000 AEs in GR and 34.31 per 1,000 AEs in FS [10]. No significant gender differences in injury incidence were observed, consistent with data from the 2008 and 2016 Olympic Games [11, 12]. 

FS had a higher injury rate than GR, a trend supported by prior research, the difference is not significant [13]. The incidence of injuries was higher among male wrestlers; however, the difference was not statistically significant. WW exhibited a higher proportion of moderate-to-severe injuries compared to the two male styles (Table 1).

Extremities sustained more severe/moderate injuries on average than the head and face region. One possible explanation is that collisions between competitors in the heat of combat can result in superficial soft tissue injuries to the face and head; these injuries are usually treatable on the mat by the on-site team and classified as minor injuries. In contrast, injuries to the extremities (joint sprains, as well as ligament and muscle strains) are more serious and may necessitate further diagnostic evaluation or treatment, and are therefore categorized as moderate injuries. Furthermore, in severe cases involving the exacerbation of pre-existing conditions or re-injury, it may result in termination of the bout.

Final matches were associated with increased injury frequency, the difference is not significant. The slightly elevated incidence numbers may be attributed to previous studies suggesting that later rounds within a match pose greater injury risk due to relative fatigue, which contributes to impaired decision-making and coordination deficits [13, 14]. Additionally, final matches involve more evenly matched opponents, which may lead to longer and more intense bouts. Neither FS nor the final matches showed elevated incidence of risk; however, the higher number of injuries per bout may require more focused medical attention from the on-site team.

Our injury severity data generally aligns with Olympic data [11, 12, 15]. We recorded mild injuries at 57.94% (Olympic range: 54.5%–84.4%), moderate injuries at 27.1% (Olympic range: 10.7%–27.3%), and severe injuries at 14.95% (Olympic range: 0%–18.2%). FS wrestlers sustained more moderate-to-severe injuries than GR (Diagram 1). Injuries to the head and face were mostly mild, whereas limb injuries were more likely to be severe (Diagram 1).

Although head and face injuries were the most common (45.79%), their incidence was lower than previous reports from major competitions (68.7%) [12]. Lacerations, bleeding, and bruising accounted for 45.7% of all injuries (12.62/1,000 AEs), consistent with previous findings (23% and 7.48/1,000 AEs; Olympic data: 54.5%) [10, 11]. Lower extremity injuries (27.1%, 7.47/1,000 AEs) also matched prior reports (12.5%–39% and 7/1,000 AEs) [10, 12]. Upper extremity injuries (18.69%) aligned with expected values (18.7%–46%), though their incidence rate (5.15/1,000 AEs) was slightly lower than the reported average (7.13/1,000 AEs) [12, 16]. Knee injuries (14.02%, 3.86/1,000 AEs) matched literature estimates of 17%–19% but exceeded prior incidence rates (1.04–2/1,000 AEs) [16–19]. 

WW showed a higher proportion of moderate-to-severe injuries, possibly attributable to biomechanical, physiological, and tactical differences. Disparities in joint stability and muscle mass may further contribute to susceptibility to injury [20]. 

Trunk and particularly spinal injuries are a concern due to their severity. Our study recorded a lower incidence of severe spinal injuries than in previous competition data (1.04/1,000 AEs vs. an expected 4.8/1,000 AEs) [10]. Strangulation injuries were rare (three cases), and all were classified as moderate. The implementation of the UWW strangulation protocol introduced in 2021 shows the effort and energy the UWW is putting into injury prevention, identifying neck, especially strangulation injuries, as injuries may be preventable with efficient intervention [19]. Such a study would be preferable; our data pool does not contain enough data for such analysis, nor do we have appropriate data from prior studies.

Despite its strengths, this study has several limitations. In this study, the documentation of injuries and the orientational diagnoses were derived from direct observations by experienced UWW medical representatives on-site. Although this method enables real-time evaluation of injury mechanisms and typical patterns, it lacks the confirmation provided by hospital diagnostics as imaging. Additionally, our study focused solely on competition settings, which restricted our ability to record injuries that occurred during training and preparation. A further limitation was the lack of follow-up on the outcomes of sustained injuries. Despite the relatively large data pool, the lack of severe injuries may bias analysis results.

Comparisons with Olympic data should be interpreted with caution due to structural differences: Olympic wrestling has a fixed number of participants (six weight classes per style, 16 wrestlers each) who qualify through rigorous selection, whereas World Championships feature a larger and more variable number of AEs and athletes with differing levels of preparation and experience.

The classification of injury severity using a three-tier system (mild, moderate, severe) and incidence rates per 1,000 AEs has only been standardized in recent years. Earlier studies relied on varying definitions and national injury registries, limiting direct comparability. Efforts should be made to further standardize injury reporting criteria to improve cross-study comparisons within wrestling and across other sports. Additionally, injury rates in competition settings, such as those in our study, should not be directly compared with training injury data, as different exposure risks apply.

### Conclusion

This study provides a comprehensive analysis of injury patterns in elite senior wrestling, revealing key risk factors related to wrestling style, gender, injury type, and anatomical location. Our findings confirm that the distribution and incidence of injuries align with prior research from high-level competitions. With a large dataset of athletic exposures, this study represents a big data pool compared to previous studies reporting competition injuries. Final matches and FS were associated with a slightly increased number of injuries; limb injuries were more severe than injuries to the head and face. Injury severity patterns aligned with previous Olympic data, with head and facial injuries being the most common but typically mild, while lower limb injuries were more often severe.

Future research should refine injury surveillance methodologies, incorporate follow-up assessments, and focus on developing sport-specific injury prevention strategies. Considering the observed trends, tailored interventions, including targeted conditioning programs and rule modifications, may help reduce injury risks. Ongoing efforts to standardize injury classification and reporting will further enhance the comparability of findings across studies and inform evidence-based medical and policy recommendations for wrestling safety.

A key consideration in the organization of medical services at competitions is that FS events are associated with a higher number of injuries; extremities are also at increased risk of more severe injuries.

## Data Availability

The data that support the findings of this study are available on request from the corresponding author. The data are not publicly available due to privacy or ethical restrictions. Permission, approval and theoretic support by the United World Wrestling.
